# Influence of habitat complexity on the prey mortality in IGP system involving insect predators (Heteroptera) and prey (Diptera): Implications in biological control

**DOI:** 10.1371/journal.pone.0264840

**Published:** 2022-03-14

**Authors:** Shreya Brahma, Dipendra Sharma, Sampa Banerjee, Goutam K. Saha, Gautam Aditya

**Affiliations:** Department of Zoology, University of Calcutta, Kolkata, India; Universitat Wien, AUSTRIA

## Abstract

Intraguild predation (IGP) is common in the freshwater insect communities, involving a top predator, intraguild prey (IG prey) and a shared prey. Influence of the habitat complexity on the prey-predator interactions is well established through several studies. In the present instance, the IGP involving the heteropteran predators and the dipteran prey were assessed in the background of the habitat complexity. The three predators *Diplonychus rusticus*, *Ranatra filiformis*, and *Laccotrephes griseus*, one intraguild prey *Anisops bouvieri* and two dipteran prey *Culex quinquefasciatus* and *Chironomus* sp. were used in different relative density against the complex habitat conditions to deduce the impact on the mortality on the prey. In comparison to the open conditions, the presence of the macrophytes and pebbles reduced the mortality of the shared prey under intraguild system as well as single predator system. The mortality of the shared prey was however dependent on the density of the predator and prey. Considering the shared prey mortality, predation on mosquito larvae was always higher in single predator system than chironomid larvae irrespective of identity and density of predators. However, for both the shared prey, complexity of habitat reduced the prey vulnerability in comparison to the simple habitat condition. Higher observed prey consumption depicts the higher risk to predation of shared prey, though the values varied with habitat conditions. Mortality of IG prey (*A*. *bouvieri*) in IGP system followed the opposite trend of the shared prey. The lower mortality in simple habitat and higher mortality in complex habitat conditions was observed for the IG prey, irrespective of shared prey and predator density. In IGP system, the shared prey mortality was influenced by the habitat conditions, with more complex habitat reducing the vulnerability of the shared prey and increased mortality of the IG prey. This implies that the regulation of the mosquitoes, in the IGP system will be impeded by the habitat conditions, with the heteropteran predators as the top predator.

## Introduction

The physical elements and the macrophytes contribute to the heterogeneity of the habitat condition in freshwater community, recognized as habitat complexity. The species interactions in the freshwater habitats are influenced by the habitat complexity, inclusive of foraging, evasion of predation [1, 2], and the prey consumption by the predators [3–7]. The movement of predators is affected by the vegetation impairing the prey search efficiency. However, contrasting examples with the habitat complexity facilitating the predation for the sit and wait predators have also been observed [2, 8–10]. For the prey, the presence of vegetation and physical structures provide refuge to evade predation [2, 11]. In several instances, due to the vegetation and the physical objects, the contact between predators and prey is altered with reduced space for interaction [12]. Contrastingly, certain studies have shown that in the complex habitat condition, the number of interacting prey and predators increases and thus the predatory-prey interactions are possibly more or less the same [13]. Thus habitat complexity influences the trophic interactions particularly the prey-predator links [14]. Irrespective of the size and shape of the water bodies and the water regime, the habitat complexity influences the freshwater community assemblages in both tropical and the temperate regions [15, 16]. The food web features, the identity of the interacting species [17–19], and the traits of the species concerned [20, 21] influence the outcome of the species interactions, all in the background of the complexity of the habitat conditions [22, 23].

The effects of the habitat complexity on the prey predator interactions involving diverse taxonomic groups have been tested in several empirical studies [5, 10, 24]. In majority instances, the prey-predator interactions are impacted by the habitat conditions and therefore appear to be strong driver of the interactions and thus the diversity and stability in the freshwater community. As observed for most of the ecological communities, the species interactions are mostly indirect [25–28], inclusive of cannibalism, omnivory, apparent competition, and intraguild predation. The extent of the outcome of these indirect interactions depends on the taxonomic identity and the relative abundance in the community [5, 29]. In view of the biological regulation of the pests [30–32] or the vector mosquitoes [33–35] the implications of the indirect interactions are significant. Biological control emphasizes the regulation for the target organism using natural enemies, which are in most instances, the generalist predators [36, 37]. Since the generalist predators are most likely to be a part of one or multiple indirect interactions, the success of the biological control would also likely to vary with the interactions. Deciphering the impact of the indirect interactions [38] in the background of the habitat complexity therefore becomes more relevant in the context of the biological control of the target organisms.

Intraguild predation is a common form of indirect interactions for most of the food webs in terrestrial and freshwater communities [39–44]. Perhaps the generalist dietary choice and abundance of similar prey and predators account for the abundance of IGP as a form of indirect interaction in food web. In IGP, the richness and the abundance of the shared prey and the intraguild prey determine the extent of variation in the predation pattern in comparison to the direct prey-predator interactions. The IGP involving beneficial and harmful insects associated with the cultivable crops is considered to be of immense importance considering the biological control as a mechanism of the regulation of the pest species [41, 45]. An alteration in the density and identity of the shared prey and the intraguild prey, as well as the alteration in the density and the identity of the predators influence the outcome of IGP interactions involving different species with taxonomic identities. This applies for regulation of both the pest species and the vector mosquitoes in a typical biological control system. In a situation where the identity and the efficacy of the IG predator are known, appropriate alteration in the predator composition may enhance the success of the biological control. Similarly, the relative density of the prey and the predators are also essential in delivering successful prey regulation. An evaluation of the prospective IGP is therefore given priority to enhance the success rate of the biological control against a target organism.

Considering IGP system under structurally complex habitat conditions, interactions among the IGP components may vary, eventually changing the fate of shared prey. In contrast to structurally simple or open habitat conditions, the antagonistic interactions between IG predator and IG prey are less likely to be occurring in complex habitat conditions that provide prey refuge [45, 46]. As a consequence, a stable coexistence of IG predator and IG prey would be possible with increased predation pressure on shared prey. Conversely, if the habitat conditions reduce the coexistence of IG predator and IG prey, reduced risk to predation will augment shared prey density. However, the antagonistic interactions between IG predator and IG prey vary with the taxonomic identity of the interacting pairs [47]. Therefore even in presence of habitat complexity, the resultant impact on the shared prey will vary depending on the identity of the IG predator. The abundance and dispersion of IG predators are influenced by the habitat structure and vegetation that may in turn influence the outcome in IGP interactions. In addition, environmental factors like habitat permanence, water depth, dispersal ability and even the availability of the alternate prey influence the outcome of IGP interactions [7].

In case of the biological control of the mosquitoes several generalist insect predators have been promoted in different larval habitats inclusive of the rice fields and bogs. Apparently, the diversity of the macroinvertebrates and the different predators are considerably high in rice fields and similar freshwater habitats that are exploited by the mosquitoes as a breeding ground [38, 48–52]. As a result, conservation biological control for the mosquitoes engaging the generalist insect predators is highlighted in these habitats. Apart from the regulation of the mosquitoes, the conservation of the insects enable sustenance of the biodiversity in the rice fields and similar freshwater habitats, supported through empirical studies in different region of the world [34, 48, 53, 54]. In India, the rice fields and similar wetlands are rich in species diversity inclusive of the mosquitoes and the water bugs, providing ample chances of direct and indirect interactions leading to the impact on the mosquito population. Several different estimates of the direct and indirect interactions can occur in the ricefield and similar habitats that may potentially interfere with the process of the mosquito regulation by the generalist insects [34]. Empirical studies have shown that the intraguild predation involving the water bugs and the dipteran larvae as prey is influenced by the density of the predator and the prey and the identity of the prey [18, 55, 56]. As a consequence, a deviation from the expected results on the biological control of mosquitoes was observed in these instances. In case of water bugs, the predation is also affected by the light and the habitat complexity [57], which may account for a barrier to predator and prey interactions or may provide refuge to the prey. In many instances, the habitat complexity may act as a barrier for the prey searching by the predators and thus may appear to be advantageous for the prey [58–60]. On the contrary, the successful regulation of the target prey may be affected due to the habitat complexity. In the present instance, we have tested the efficacy of the water bugs engaged in the intraguild predation in the different context of the habitat complexity. The observations may aid in the understanding of the extent of the regulatory effect imposed by the water bugs on the mosquito population in the rice fields. While the habitat complexity is known to influence the species interactions including prey-predator interactions, the impact on the indirect interactions like the intraguild predation are little explored. Particularly, when the generalist predators are common in several of the freshwater wetlands, the possibility of indirect interactions are considerably high. Besides, the habitat complexity due to the weeds and the sediment conditions may lead to additional constraint for the prey-predator interaction. The results of the present study will enable us to explore the possible role of the habitat complexity on the intraguild interactions involving mosquito as target prey and the heteropterans as the predator.

## Material and methods

### Study organisms

#### Intraguild predators

The three water bug species, *Diplonychus rusticus* Fabricius, 1781 (Heteroptera: Belostomatidae), the water scorpion *Laccotrephes griseus* (Guerin-Manevilli, 1844) (Heteroptera: Nepidae) and the water stick insect *Ranatra filiformis* Fabricius, 1790 (Heteroptera: Nepidae) were considered as the top predators (intraguild predator) in the present study. While the water bug *D*. *rusticus* was active predator and hunt prey actively, the water scorpion *L*. *griseus* and water stick insect *R*. *filiformis* were sit and wait predators [61–64]. The three species are common in the different freshwater bodies in West Bengal, India, consuming wide range of the prey species including the chironomid and the mosquito larvae. Observations [65–69] on the morphological features of these predators and the habitat preferences indicate that these predators are common in the different wetlands and share mosquito and the chironomid larvae as shared prey. The collection of these predators was made from the different water bodies around the Ballygunge Science College campus, Kolkata, India using an insect net of 200 μm mesh size fitted with a long wooden handle [70]. The insect net was moved through the littoral zone of the water bodies with moderate vegetations and the collected specimens were brought to the laboratory for the rearing and maintenance. The average body lengths of adult *D*. *rusticus*, *R*. *filiformis*, *L*. *griseus* were 16.4 mm (range, 15–18 mm),52.01 mm (range, 44.8–58 mm), 26.20 mm (range, 21–32 mm) respectively. The body length was measured from the tip of the rostrum to the end of the abdomen. For the predators the body length corresponded to the adult morphs [71].

#### The intraguild prey

The backswimmer, *Anisops bouvieri* Kirkaldy, 1904 (Heteroptera: Notonectidae) was considered as the intraguild prey in the IGP system. Also, it was considered as a predator in the single predator experiments using mosquito and chironomid larvae as the prey. The backswimmers are common in almost all type of freshwater habitats including ponds, lakes, rivers, streams, stagnant waters in peatlands, marshes or swamps, though less available in vegetated area, swimming close beneath the water surface. Empirical studies suggest that the backswimmers are generalist predators and the prey item includes both mosquito and chironomid larvae. The average body length of the adult *A*. *bouvieri* was 6.32 mm (range, 5.8–7.2 mm) measured from tip of the rostrum to the end of the abdomen.

#### Shared prey

The larval stage of the mosquito *Culex quinquefasciatus* Say, 1823 (Diptera: Culicidae) and the chironomid, *Chironomus* sp. (Diptera: Chironomidae) were considered as the shared prey in the present experiment. The instar IV stage larvae of mosquito and chironomid midges were considered as the prey for the experiments. Following collection of the larvae of the chironomid and the mosquito, segregation and selection of the desired size classes were done for the experiment as stated below.

### Collection and maintenance of study insects

#### The insect predators

The collection of the predatory insects was made from different ponds and temporary pools in and around Kolkata, India. The aquatic bodies were 100–400 m^2^ in area, 50–100 cm in depth, rectangular to oval in shape with sparse vegetation upto a level of 50 cm from the bank. The vegetations included *Pistia stratiotes*, *Jussiaea repens*, *Alternanthera philoxeroides*, *Lemna major*, *Sagittaria* sp., *Nymphoides* sp., *Ipomoea aquatica* in varying proportion. During collection small indigenous fishes like *Colisa fasciata*, *Punctius ticto* and *Aplocheilus panchax* were also encountered as representative fish species. Collected insects were brought to the laboratory in separate jars and emptied in water filled aquarium (38 x 36 x 36 cm). The predatory insects, collected from different ponds, separated species wise and maintained in the aquarium at a density of 25 individuals per 35 L of pond water with few specimens of *Lemna minor*, *Pistia stratiotes* and *Vallisneria spiralis* as refuge. Mosquito and chironomid larvae and tubificid worms collected from the same habitats and adjacent sewage drains were provided as food *ad libitum*. The insect predators were maintained in the laboratory for at least seven days before using them for experiment. The collection was continued as per the requirements of the experiment and the insects were maintained in a similar way as stated above.

#### The shared prey

The mosquito *C*. *quinquefasciatus* larvae were collected from the sewage drains in and around Ballygunge Science College campus, University of Calcutta, Kolkata. The collected larvae were brought to the laboratory and placed in an enamel tray (45 x 30 x 7.5 cm) for segregation of the IVth instar larvae (5.1–6.0 mm in length, IV instar; 1.9–2.1 mg in weight) to be used in the experiments. The rest of the larvae were retained in the enamel tray and were provided with Laviest capsules^®^ (Franco-Indian Pharmaceuticals, Mumbai, India) as source of food to grow to instar IV and were subsequently used in the experiments. The pupae collected if any were killed by drying. The collections of larvae were continued time to time as per the requirement of the experimental trials. For segregation and maintenance of chironomid larvae, the sediment collected was poured within enamel trays (45 x 30 x 7.5 cm) containing sewage drain and tap water (1:1:: v/v) and was placed under a light source. The set-up was left undisturbed for 3–4 hrs to allow the sediment to settle down and the chironomid larvae to emerge out from the sediment and cling to the sides of the tub. Subsequently, the larvae were separated with a pipette; the larger ones (> 20mm in length, 3.3–5.1 mg in wet weight) sorted and placed within smaller trays with little sediment from where they were used for the experimental purposes. In course of collection of study insects, the relative numbers in each sample were recorded and length and weight were measured for selected specimens.

### Experimental design

The experimental protocol followed a complete randomized block design using 4 levels of habitat complexity, two levels of IG predator density and two levels of shared prey density. Three different species (*D*. *rusticus*, *R*. *filiformis* and *L*. *griseus)* were used separately as IG predators against two different species of shared prey (chironomid larvae and instar IV larvae of *C*. *quinquefasciatus* larvae). To determine the effects of IGP with four different habitat conditions, two different experiments were carried out with each predatory insect species, first as single predator followed by multiple predators constituting the IGP system.

#### Experiment 1

In the laboratory, experiments were carried out in eighteen glass aquarium (38 x 36 x 36 cm), each of 35 l water capacity. Each of single predator species (*D*. *rusticus*, *R*. *filiformis*, *L*. *griseus*, *A*. *bouvieri*)of a particular density (2 or 4 for three IG predators and 10 for *A*. *bouvieri*) was provided with two different densities (50; low and 200; high individuals) of prey species (mosquito or chironomid larvae)under 4 levels of habitat complexity and prey consumption was noted for 24 hours. In this single predator experiment *A*. *bouvieri* is used as a predator without any IG predator.

#### Experiment 2

In the second set of experiments two different predators (IG predator and IG prey) were provided with either mosquito or chironomid larvae as a prey, where *A*. *bouvieri* was considered as IG prey. The density of IG prey remained constant (10 individuals) while the shared prey was provided with two different densities—50 (low) and 200 (high) individuals and two levels of density– 2 (low) or 4 (high) of IG predators. In this instance, *A*. *bouvieri* is used as IG prey, where it is vulnerable to the IG predator but can consume the shared prey.

**Fig 1:**
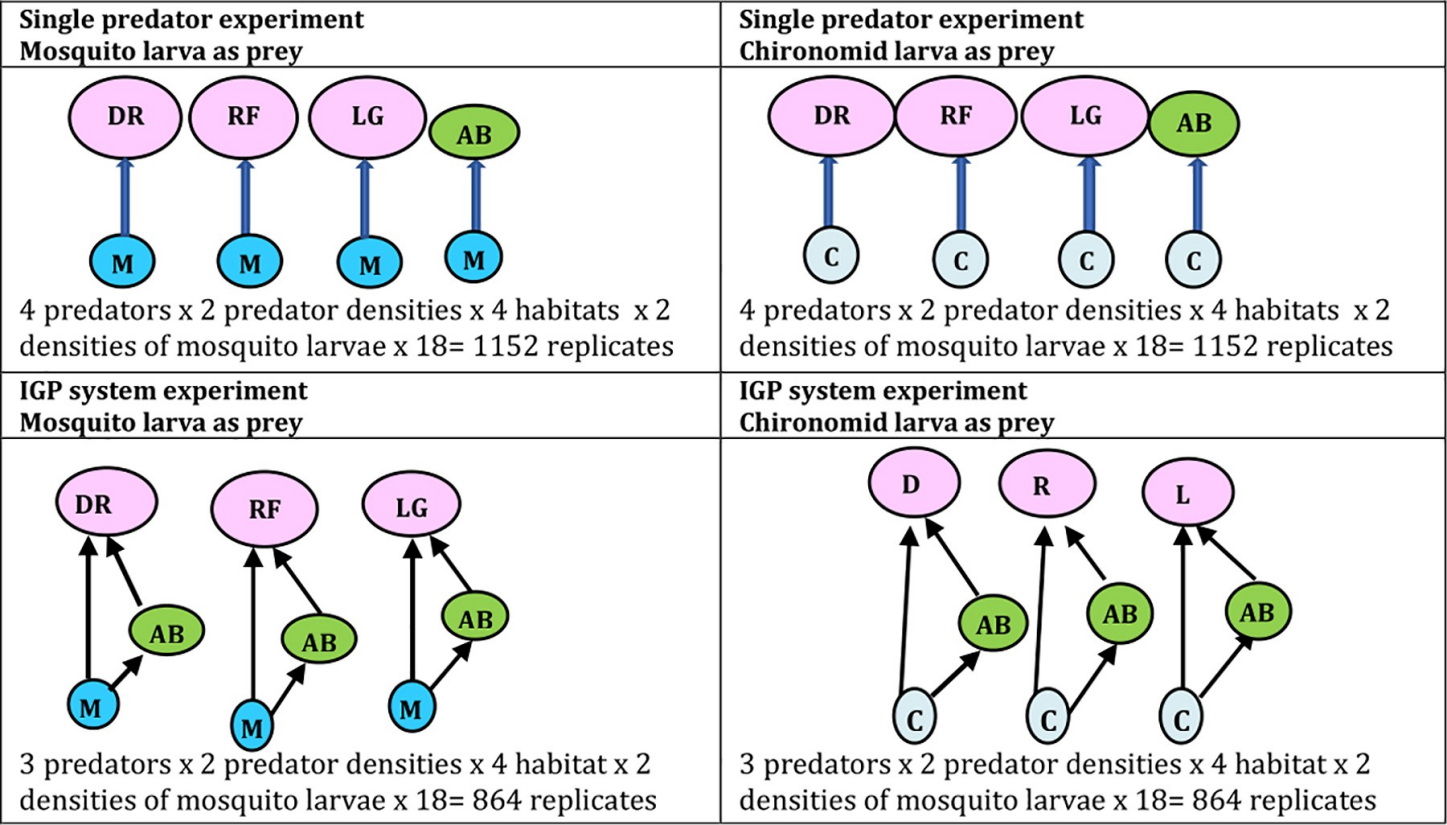
The portrayal of the interactions among the insect predator and prey involving the intraguild predation (IGP) system. As a shared prey both chironomid and mosquito larvae (Diptera) are consumed by the insect predators (Heteroptera) individually, as well as a part of the IGP system. Initial observations on single prey predator experiments were followed by the intraguild predation experiments under the varied habitat conditions represented in below.

Both the single predator and multiple predator experiments were carried out with four different habitat conditions separately. Three complex habitat conditions inclusive of the pebbles, vegetation and pebbles and vegetations were considered along with open condition without pebbles and vegetation. The vegetation condition was constructed using the sticks of *Ipomoea aquatica*, floating *Pistia stratiotes* and *Vallisneria spiralis* (Fig 1), while the small stones of varied diameter (used in aquarium) were used to construct the pebble condition. Using the four habitat conditions the experiments were conducted to deduce the effects of–

1. Habitat conditions on both the single predator and the IGP system
2. Differences in IGP systems with IG predators of different taxonomic identity
3. Differences in the prey mortality under different habitat conditions, both in the single predator and IGP system.

**Fig 2:**
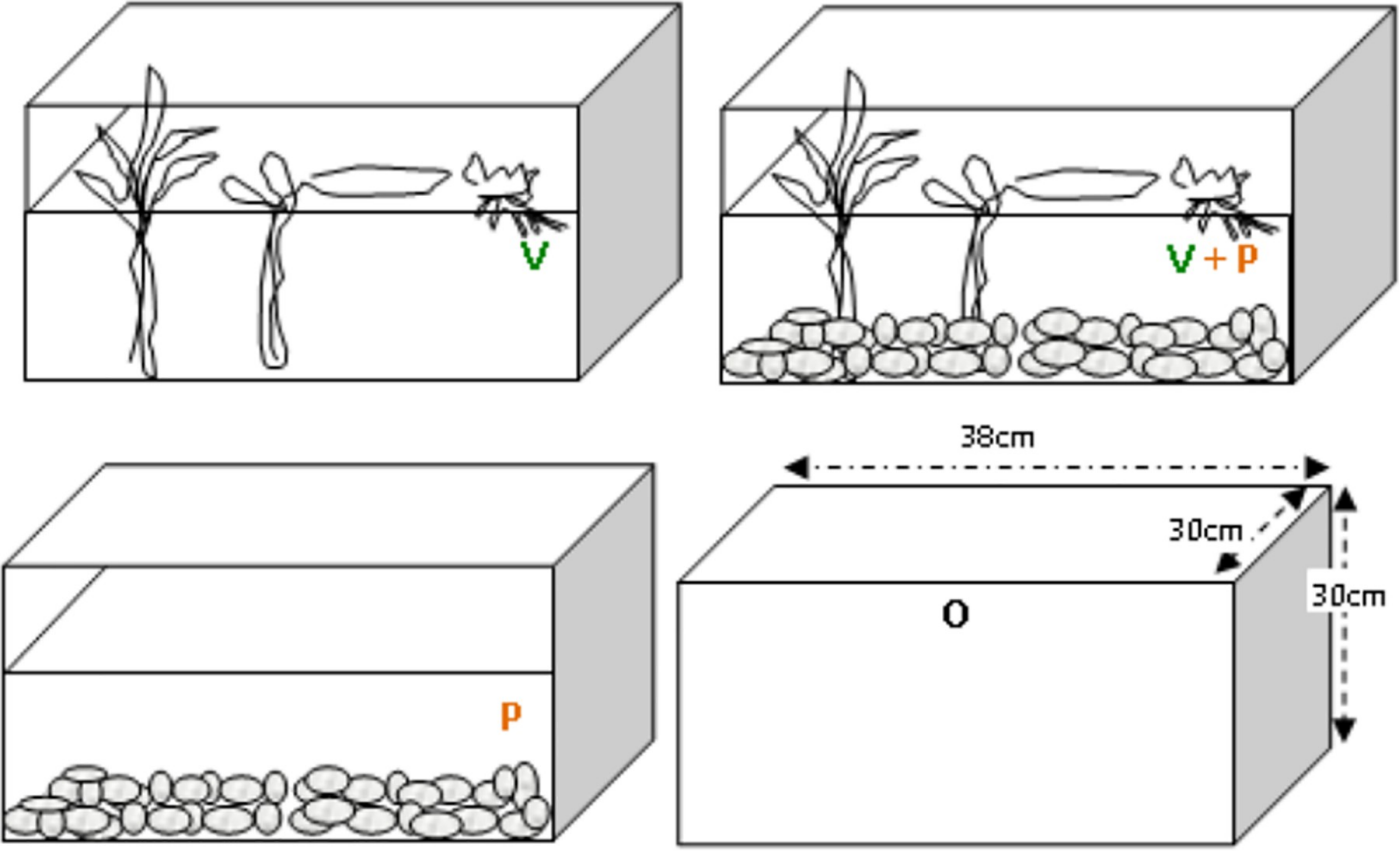
Illustration of the habitat conditions used to evaluate the influence of the habitat complexity on the IGP system. Four different habitat conditions in the glass aquaria using vegetations (V), vegetations and pebbles (V+P), pebbles (P) and open conditions (O) were created in the glass aquarium. The condition (O) is used as simple condition reflects no habitat complexity in contrast to the rest three. Glass aquarium: Experimental mesocosm Water– 35L tap: pond; (pH– 7.6 to 8.2; temperature observed 29–32°C) Observation on predation was measured for 24 h. for each replicate.

The prey and predator combinations and the total number of replicates considered in the experiments are mentioned in Fig 1, while the arrangement of the experimental containers is shown in Fig 2. The data on the mortality of the prey (for single predator experiments) or shared prey (for IGP system) was recorded and applied to the multiplicative risk model to test the increase or decrease in the risk in mortality of the shared prey in IGP system.

### Data analysis

#### Prey consumption

The data on the shared prey (mosquito larvae or chironomid larvae) consumption and the data on the IG prey consumption (*Anisops* sp.) were subjected to the logistic regression analysis, separately. In case of the shared prey consumption, the predator combination (single predator system and the IGP system), the shared prey density (low or high), the predator density (low or high), the habitat conditions (open, pebble, vegetation, and vegetation and pebbles) were considered as the explanatory variables and the prey consumption as the response variable. Likewise, the shared prey density, the predator density and the habitat conditions were considered as the explanatory variables against the IG prey as the response variable. The analyses were carried out for the three top predators separately, for the shared prey consumption and the corresponding IG prey consumption. In the logistic regression, based on the principles of the binomial generalized linear model (GLM) with logit link function, the prey consumption was assumed to comply binomial (n, p) distribution. Here, n represents the number of replicates for each combination of explanatory variables, while, p is the probability parameter representing the linear combination of the explanatory variables. Using a logit link function, the parameters of the regression equation were measured through the maximum likelihood methods using XLSTAT [72].

The logistic regression was of the form, (y) = 1/ (1+ exp(–(a +b_i_x_i_ +. . . . + b_n_x_n_))), where x represents the explanatory variable (i to n numbers), y represents the response variables (shared prey consumption or the IG prey consumption). The response variable was weighted against the respective prey density provided at the initiation of the experiment to qualify for the analysis as binary (sum) proportion values A Chi square value (Wald’s Chi-square) was used to deduce the significance of the estimated parameters of the model that included predator density, prey density, predator combination and the habitat conditions for the shared prey consumption. Similarly, for the IG prey consumption, the significance of the estimated parameters was deduced for the prey density, predator density and habitat conditions. In the present regression model, the explanatory variables are considered independently, and not the interactions among them.

### Model assessment

#### The model

To assess the independence between predator effects, the observed predation rate was compared with the predicted values following the ‘multiplicative risk model’ [73–76]. This model predicts combined risk to the prey when both IG predator and IG prey were present but their effects were independent. Specifically, this model predicts that the expected proportion of prey killed by predator species A and B together (*p*_AB_) is:

$$p_{AB}=p_{A}+p_{B}‐p_{A}p_{B}$$

where *p*_AB_ is the predicted combined consumption for a particular initial prey density; *p*_A_ is the probability of being consumed by predator species A in isolation, and *p*_B_ is the probability of being consumed by predator species B in isolation over a 24 hrs. period of exposure. The *p*_A_*p*_B_ term in the model accounts for prey removal by both predators.

#### Habitat effect assessment

To compare the effects of habitat complexity on the vulnerability of the IG prey, a coefficient ‘*k*’ was used to represent the proportional difference between open and complex habitat conditions as shown in the following equations-

*k*_v_ = PM_o_/ PM_v_, for habitat conditions with vegetation only

*k*_*p*_ = PM_o_/PM_p_, for habitat conditions with pebbles only

*k*_*v+p*_ = PM_o_/PM_v+p_, for habitat conditions with vegetation and pebbles present together. A value greater than 1 would indicate that the prey mortality was higher in simple conditions, than in complex conditions, while a value of less than 1 would indicate greater prey mortality in complex conditions. A two-tailed t-test [77] was applied to justify whether the values are significantly different from when compared against the simple habitat conditions.

The outline of the experiments carried out along with the number of replicates and data analysis are shown in (**S1 Table 1 in S1 File**).

In the present instance, to carry out the experiments, no specific permissions were required for any locations / activities for the study. Besides, the experiments in the study did not engage endangered or protected species. The collection and maintenance of the study insects followed the rules and regulations of the Institutional Animal Ethical Committee, Department of Zoology, University of Calcutta, Kolkata, India.

## Results

### Single predator experiment

In all the experimental conditions, the shared prey mortality was observed in varying degree with reference to the density levels of the predators and the prey. When compared for the four different habitat conditions, the prey vulnerability varied for each of the prey and predator species combinations. Apparently, the prey vulnerability was a function of the predator identity and relative abundance of the concerned predatory insect. In both the single predator and IGP systems, the prey mortality was influenced by the complexity of the habitat conditions. The general trend, irrespective of density and identity of prey and predators, was that, under simple habitat condition, the prey mortality was consistently higher than the other three levels of complexity (pebbles and vegetation alone or separately present) (**S1 Tables 2 and 3 in S1 File**). At a low prey density (50 individuals), the vulnerability of mosquito and chironomid larvae was higher in simple habitat conditions and oppositely lower in complex habitat conditions (both vegetation and pebbles when present in the habitats). On a comparative scale the predators consumed higher number of mosquitoes than chironomid larvae when present separately as individual predator. Although density effects were evident for chironomid and mosquito larvae, the habitat conditions appeared to be more determining factor.

For all the predators, the prey consumption was density dependent and differed significantly among the predator species. *D*. *rusticus* consumed at a greater amount than the other two predators when present as individual predators. For *A*. *bouvieri*, the trend in mosquito and chironomid larvae consumption remained similar to the rest of the heteropteran predators with highest consumption in simple conditions and lowest in complex conditions with vegetation and pebbles (S3 Table in S1 File). The results of the logistic regression indicated that the shared prey mortality was influenced significantly (P < 0.001) by the prey density, predator density, predator combinations (either single or in IGP) and the habitat conditions, for both mosquito and the chironomid as prey against the three heteropteran IG predators (Table 1) except for the predator density in case of *D*. *rusticus*, and prey density in *L*. *griseus*, which were insignificant. Similar observations were also made for the IG prey mortality (Table 2), with the prey density and predator density and habitat combinations significantly (P < 0.001) contributing to the IG prey mortality in the IGP system.

**Table 1:** (a) The logistic regression equations representing the variations in the shared prey consumed (preycon) by the IG predators against the prey density (preyden), predator density (predden) and predator combination (predcomb) and habitat complexity (habitat) as explanatory variables. Predator combinations were (i) only IG prey and shared prey ii) both IG predator and IG prey with shared prey iii) only IG predator and shared prey. The level of significance assumed to be 0.025. The prey and predator combinations are shown in the suffix. (b) Significant values of the parameters of the model (in bold) were deduced through the Wald’s Chi-square test represented below. Here, the prey predator combinations were, mosq–Mosquito larvae, rus–*D*. *rusticus*, Chiro–chironomid larvae, ran- *R*. *filiformis*, lacco–*L*. *griseus*.

**(a)**Logistic regression of prey-predator combination. Significant at least at 0.025 level.							
preycon_Mosq-rus_ = 1 / (1 + exp(-(2.964–0.547*preyden+0.330*predden-0.793*predcomb-0.476*habitat)))							
preycon_Mosq-ran_ = 1 / (1 + exp(-(1.515–0.052*preyden+0.269*predden-0.665*predcomb-0.480*habitat)))							
preycon_Mosq-lacco_ = 1 / (1 + exp(-(2.487–0.562*preyden+0.403*predden-0.592*predcomb-0.548*habitat)))							
preycon_Chiro-rus_ = 1 / (1 + exp(-(1.964–0.334*preyden+0.191*predden-0.611*predcomb-0.368*habitat)))							
preycon_Chiro-ran_ = 1 / (1 + exp(-(0.560–0.280*preyden+0.624*predden-0.543*predcomb-0.361*habitat)))							
preycon_Chiro-lacco_ = 1 / (1 + exp(-(1.814–0.493*preyden+0.400*predden-0.535*predcomb-0.362*habitat)))							
**(b)**							
**Mosq-rus**	**Value**	**SE**	**Wald χ** ^**2**^	**Chiro-rus**	**Value**	**SE**	**Wald χ** ^**2**^
Intercept	**2.964**	0.044	4557.052	Intercept	**1.964**	0.042	2165.318
Preyden	**-0.547**	0.017	1092.543	preyden	**-0.334**	0.016	428.433
predden	**0.330**	0.013	615.448	predden	**0.191**	0.013	212.878
predcomb	**-0.793**	0.008	8832.822	predcomb	**-0.611**	0.008	5564.313
habitat	**-0.476**	0.006	6044.175	habitat	**-0.368**	0.006	3815.284
**Mosq-ran**				**Chiro-ran**			
Intercept	**1.515**	0.043	1213.918	Intercept	**0.560**	0.044	161.380
preyden	**-0.052**	0.017	9.539	preyden	**-0.280**	0.017	269.000
predden	**0.269**	0.014	393.983	predden	**0.624**	0.014	1934.644
predcomb	**-0.665**	0.009	6050.559	predcomb	**-0.543**	0.009	3805.720
habitat	**-0.480**	0.006	5887.774	habitat	**-0.361**	0.006	3169.015
**Mosq-lacco**				**Chiro-lacco**			
Intercept	**2.487**	0.043	3294.710	Intercept	**1.814**	0.042	1886.404
preyden	**-0.562**	0.016	1168.354	preyden	**-0.493**	0.016	946.564
predden	**0.403**	0.013	905.213	predden	**0.400**	0.013	938.073
predcomb	**-0.592**	0.008	5013.078	predcomb	**-0.535**	0.008	4350.008
habitat	**-0.548**	0.006	7812.272	habitat	**-0.362**	0.006	3750.707

**Table 2:** The logistic regression on the IG prey consumed (anicon) against shared prey density (preyden), predator density (predden) and habitat complexity (habitat) as the explanatory variables. The level of significance is 0.025. (b) Significant values of the parameters of the model (in bold) were deduced through the Wald’s Chi-square test represented below. Here, the prey predator combinations were, mosq–Mosquito larvae, rus–*D*. *rusticus*, Chiro–chironomid larvae, ran- *R*. *filiformis*, lacco–*L*. *griseus*.

**(a)**Logistic regression of IGP system. Significant at least at 0.025 level.							
anicon_Mosq-rus_ = 1 / (1 + exp(-(-2.023–0.299*preyden-0.037*predden+0.629*habitat)))							
anicon_Mosq-ran_ = 1 / (1 + exp(-(-2.614–0.357*preyden+0.780*predden+0.554*habitat)))							
anicon_Mosq-lacco_ = 1 / (1 + exp(-(-3.699–0.167*preyden+0.705*predden+0.739*habitat)))							
anicon_Chiro-rus_ = 1 / (1 + exp(-(-0.626–0.519*preyden-0.416*predden+0.460*habitat)))							
anicon_Chiro-ran_ = 1 / (1 + exp(-(-0.308–0.478*preyden-0.453*predden+0.425*habitat)))							
anicon_Chiro-lacco_ = 1 / (1 + exp(-(-1.910–0.227*preyden+0.254*predden+0.416*habitat)))							
**(b)**							
**Mosq-rus**	**Value**	**SE**	**Wald χ** ^**2**^	**Chiro-rus**	**Value**	**SE**	**Wald χ** ^**2**^
Intercept	**-2.023**	0.214	89.100	Intercept	**-0.626**	0.203	9.541
preyden	**-0.299**	0.086	12.191	Preyden	**-0.519**	0.084	38.007
predden	-0.037	0.086	0.183	Predden	**-0.416**	0.084	24.439
habitat	**0.629**	0.041	240.357	Habitat	**0.460**	0.039	141.937
**Mosq-ran**				**Chiro-ran**			
Intercept	**-2.614**	0.208	157.285	Intercept	-0.308	0.195	2.499
preyden	**-0.357**	0.082	18.913	Preyden	**-0.478**	0.081	34.889
predden	**0.780**	0.083	89.175	Predden	**-0.453**	0.081	31.280
habitat	**0.554**	0.038	211.930	Habitat	**0.425**	0.037	132.663
**Mosq-lacco**				**Chiro-lacco**			
Intercept	**-3.699**	0.236	246.060	Intercept	**-1.910**	0.206	85.672
preyden	-0.167	0.088	3.580	Preyden	**-0.227**	0.082	7.573
predden	**0.705**	0.089	62.392	Predden	**0.254**	0.083	9.479
habitat	**0.739**	0.043	298.433	Habitat	**0.416**	0.038	121.017

### IGP system experiment

**Fig 3:**
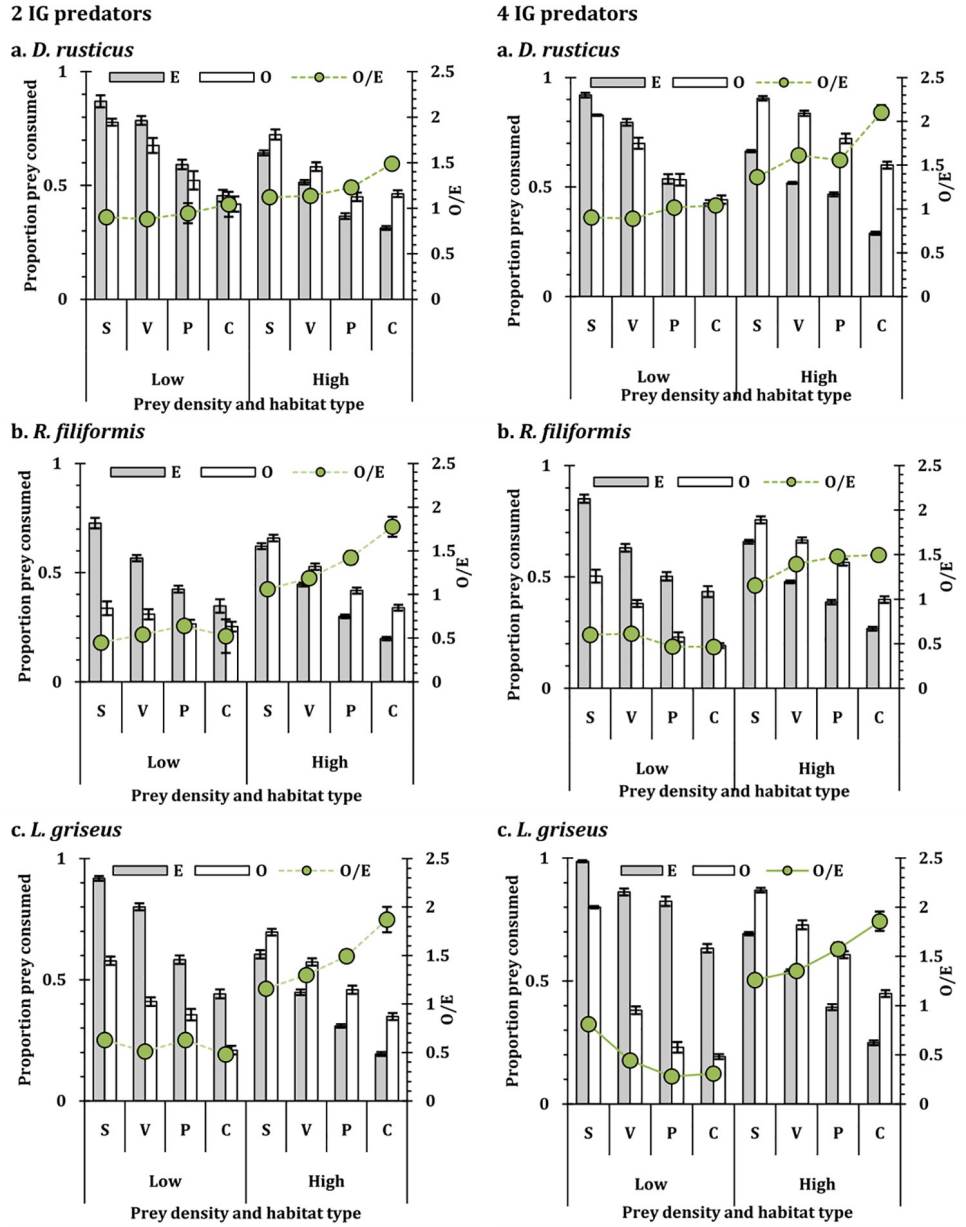
The observed (unfilled bar) and expected (filled bar) number (mean ± SE) of mosquito larvae mortality at low (50 individuals) and high (200 individuals) density in heteropteran IGP using *D*. *rusticus* (D), *R*. *filiformis* (R), and *L*. *griseus* (L) separately as IG predators and ten individuals of *A*. *bouvieri* as IG prey at low (2 individuals) and high (4 individuals) of IG predator density. The secondary y-axis represents Observed/Expected value (O/E) of shared prey mortality. S = Simple; V = Only macrophytes; P = only pebbles; C = Complex; E = Expected value; O = Observed value; O/E = Observed/ expected value.

**Fig 4:**
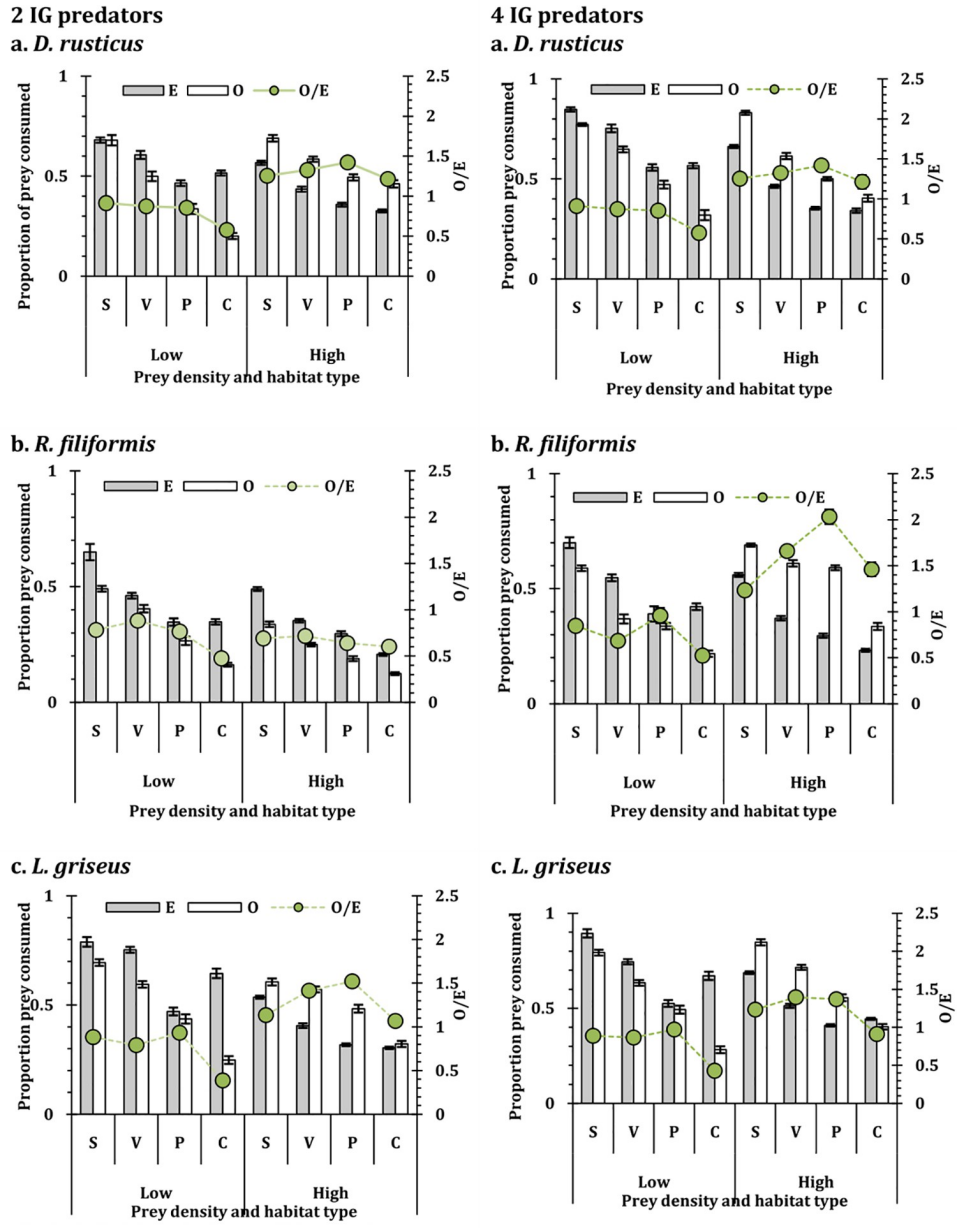
The observed (unfilled bar) and expected (filled bar) number (mean ± SE) of chironomid larvae mortality at low (50 individuals) and high (200 individuals) density (primary y-axis) in heteropteran IGP using *D*. *rusticus* (D), *R*. *filiformis* (R), and *L*. *griseus* (L) separately as IG predators and ten individuals of *A*. *bouvieri* as IG prey at low (2 individuals) and high (4 individuals) of IG predator density. The secondary y-axis represents Observed/Expected value (O/E) of shared prey mortality. S = Simple; V = Only macrophytes; P = only pebbles; C = Complex; E = Expected value; O = Observed value; O/E = Observed/ expected value.

The mosquito and chironomid prey mortality were influenced by the habitat conditions in the IGP system, at both the densities of IG predator (2 and 4) (**S1 Table 4 in S1 File**). The associated mortality of the IG prey in the IGP system was also influenced by the habitat conditions (**S1 Table 5 in S1 File**). In all instances, irrespective of shared prey density, IG prey mortality was high in complex habitat conditions and low in simple habitat conditions. It was apparent that for both the shared prey, complexity of habitat reduced the risk of being consumed by the predators while simple conditions increased the risk to predation (Figs 3 and 4). The observed prey consumption was consistently higher than expected for all the instances, though the values were conversely higher for the low shared prey density. This reflects that the risk to predation increased for the shared prey though the values varied with the habitat conditions.

**Fig 5:**
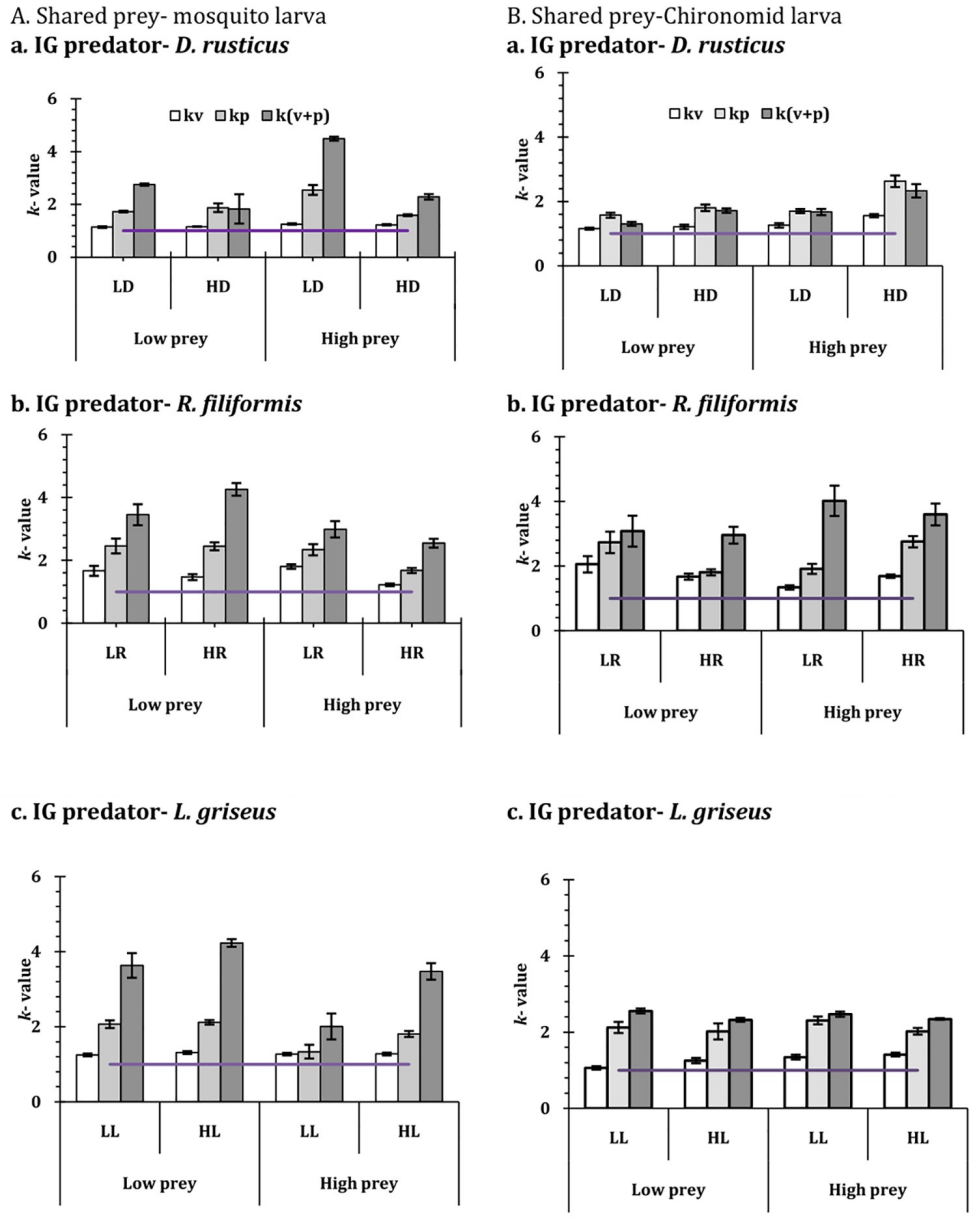
Mortality of shared prey (**A—mosquito larvae, B- chironomid larvae**) in **IGP system** in complex habitat conditions (v–vegetation, p–pebbles and v+p—vegetation and pebbles) against simple conditions expressed as a ratio (*k–*value, mean ± SE) for the three IG predators in two density (L– 2 individuals and H—4 individuals) under low and (50 individuals) high (200 individuals) prey density. The horizontal lines in each graph represents the reference value of 1, equivalent to the value of no difference between the complex habitat condition and open condition.

**Fig 6:**
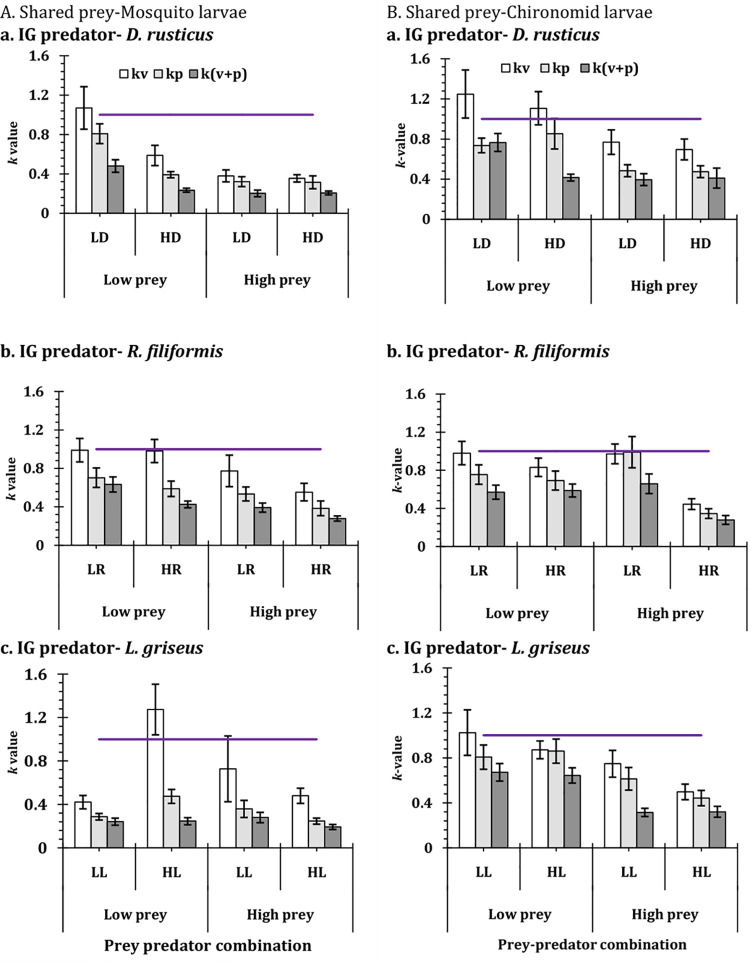
Mortality of IG prey (*A*. *bouvieri*) in presence of different shared prey (**A. mosquito larvae** and **B. chironomid larvae**) in complex habitat conditions (v–vegetation, p–pebbles and v+p—vegetation and pebbles) against open conditions expressed as a ratio (*k–*value, mean ± SE) for the three IG predators, in **IGP system**, in two density (L– 2 individuals and H—4 individuals) under low and (50 individuals) and high (200 individuals) prey density. The horizontal lines in each graph represents the value of 1.

**Fig 7:**
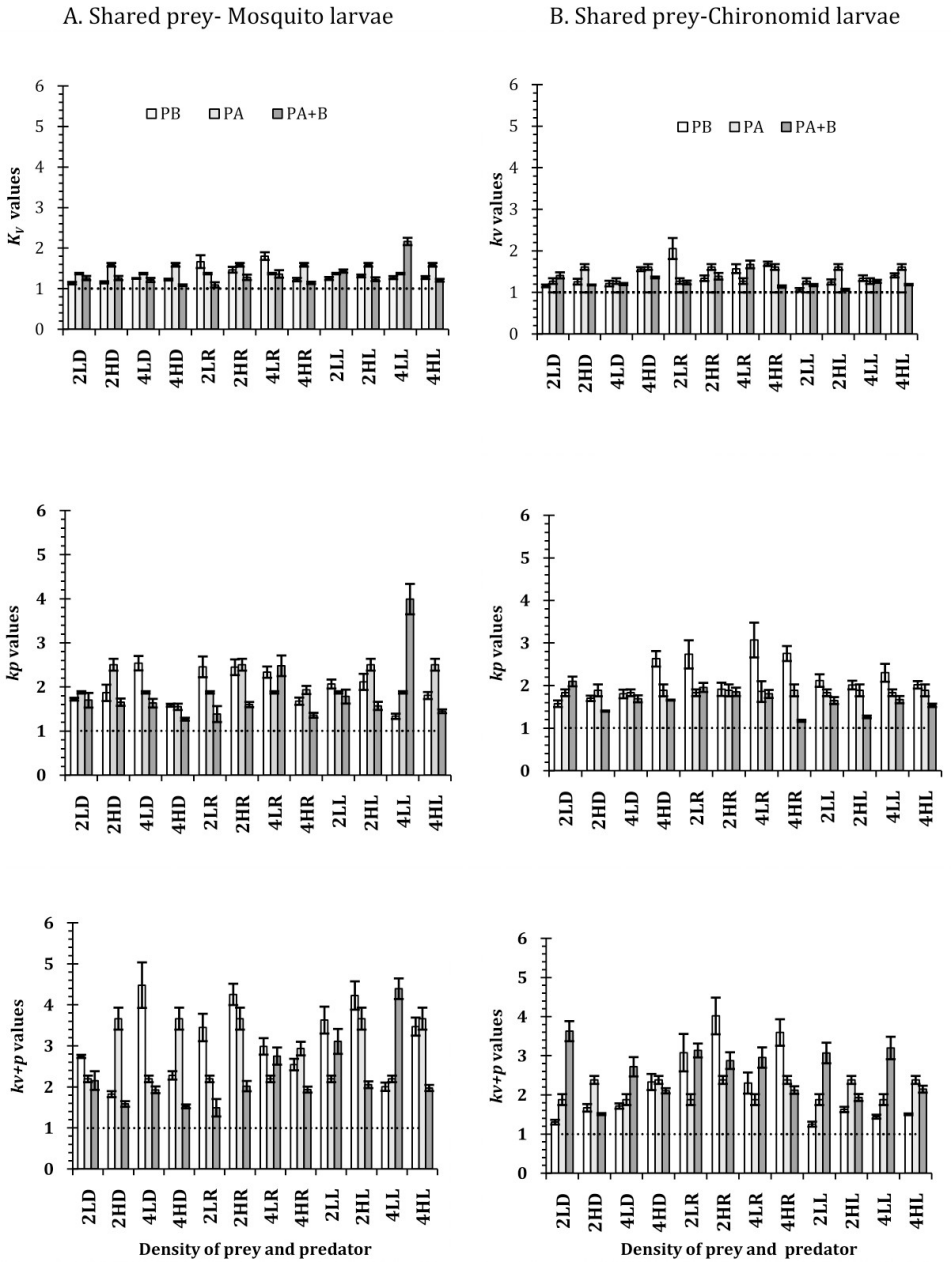
The ‘multiplicative risk model’ measures (*p*_A_, *p*_B_, *p*_A+B_) for the IGP system with different shared prey (**A. mosquito larvae and B. chironomid larvae**) in complex habitat conditions (v–vegetation, p–pebbles and v+p—vegetation and pebbles) against open conditions expressed as a ratio (*k–*value, mean ± SE) for the three IG predators (S—*D*. *rusticus*, R—*R*. *filiformis* and L—*L*. *griseus*), in two density (L– 2 individuals and H—4 individuals) under low and (50 individuals) and high (200 individuals) prey density. The dashed lines in each graph represent the reference value of 1, equivalent to the value of no difference between the complex habitat condition and open condition.

The effects of habitat conditions on the prey mortality are substantiated through the values of *k* factor that were consistently greater than 1 for all the combinations of shared prey and predator species density and identity (Fig 5). In contrast the *k* factors were consistently less than 1 for the mortality of IG prey (Figs 6 and 7) except for the high predator density and low prey density combination of *L*. *griseus*, and the low prey density of *D*. *rusticus*. For both instances the *k* factor were significantly different from 1 (Tables 3 and 4) for all instances of prey and predator combinations. The pattern of mortality of the shared prey and the IG prey under simple and complex conditions appear to be complementary to one another. High shared prey mortality is associated with low IG prey mortality and vice versa (**S1 Tables 4 and 5 in S1 File**). The expected values of the models too exhibited considerable variations with respect to the complex and simple habitat conditions (Fig 7). As shown in the figures, for all the combinations of predators and prey, the *k*-values increased with the levels of complexity. In comparison to the situation when the macrophytes were present as element of complexity, the values of *k* increased for the conditions, when the pebbles were present as well as when macrophytes and pebbles were present as the factors of complexity (Fig 7). Pertinently, the results represent that the increase in the complexity leads to the decreased consumption of the shared prey (chironomid or mosquito larva) and more impact on the IG prey by the top predator.

**Table 3:** The results of t-test using mosquito larvae as shared prey in the IGP system with different habitat conditions (v–vegetation, p–pebbles and v+p—vegetation and pebbles) against open condition.

Shared prey	Predator density	Prey density	Predator species	*k*- value		
				*k*_v_	*k*_p_	*k*_(v+p)_
**Mosquito larvae**	**2**	**50**	AB	21.176	32.758	14.961
			DR	4.564	23.375	44.648
			RF	4.202	6.182	7.337
			LG	6.913	10.492	8.018
			AB+DR	**2.395**	4.209	5.077
			AB+RF	**1.405**	**2.132**	**2.322**
			AB+LG	10.975	4.970	6.954
		**200**	AB	13.097	11.226	9.954
			DR	5.883	4.684	11.242
			RF	6.575	8.155	12.542
			LG	9.358	6.125	9.356
			AB+DR	4.981	7.916	8.581
			AB+RF	4.179	10.197	8.126
			AB+LG	5.003	6.170	12.698
	**4**	**50**	AB	21.176	32.758	14.961
			DR	28.007	9.400	6.275
			RF	8.477	10.465	9.901
			LG	7.398	5.584	9.901
			AB+DR	4.246	6.441	11.299
			AB+RF	3.880	6.329	8.358
			AB+LG	12.874	8.682	13.520
		**200**	AB	13.097	7.720	9.954
			DR	10.125	17.119	12.496
			RF	5.425	8.297	11.010
			LG	7.776	10.121	11.263
			AB+DR	4.400	8.242	11.257
			AB+RF	4.891	6.548	13.027
			AB+LG	6.065	10.500	13.186

All the t- values (df = 17; two-tailed) are significant at P<0.05 level except those in bold. Here AB is the IG prey *A*. *bouvieri*, DR–*D*. *rusticus*, RF–*R*. *filiformis*, and LG–*L*. *griseus*.

**Table 4:** Results of the t-test using chironomid larvae as shared prey in the IGP system with different habitat conditions (v–vegetation, p–pebbles and v+p—vegetation and pebbles) against open condition.

Shared prey	Predator density	Prey density	Predator species	k- value		
				k_v_	k_p_	k_(v+p)_
**Chironomid larvae**	**2**	**50**	AB	3.943	10.457	6.341
			DR	4.719	7.435	5.110
			RF	4.179	5.240	4.351
			LG	**1.267**	7.609	3.956
			AB+DR	5.615	9.637	10.272
			AB+RF	5.200	8.921	11.983
			AB+LG	6.483	7.914	7.874
		**200**	AB	8.679	6.371	13.542
			DR	3.742	11.095	7.010
			RF	5.074	5.874	6.439
			LG	4.025	9.958	9.190
			AB+DR	17.390	29.215	19.825
			AB+RF	4.841	8.597	8.792
			AB+LG	**2.822**	8.680	10.457
	**4**	**50**	AB	3.943	10.457	6.341
			DR	3.140	7.950	10.565
			RF	5.315	5.069	4.840
			LG	4.989	6.185	8.814
			AB+DR	7.752	8.469	6.964
			AB+RF	7.253	8.544	7.519
			AB+LG	7.211	7.939	7.669
		**200**	AB	8.679	6.371	13.542
			DR	11.456	8.976	6.456
			RF	12.962	9.954	7.679
			LG	8.231	11.693	23.128
			AB+DR	16.964	56.244	16.767
			AB+RF	4.991	7.092	11.616
			AB+LG	11.763	13.487	13.080

All the t- values (df = 17; two-tailed) are significant at P<0.05 level except those in bold. Here AB is the IG prey *A*. *bouvieri*, DR–*D*. *rusticus*, RF–*R*. *filiformis*, and LG–*L*. *griseus*.

## Discussion

Prey predator interactions in aquatic communities are influenced by habitat conditions, as evident from studies on insect predators with varied taxonomic identity [5, 46, 78–83]. The vegetation and other physical structures that constitute the complexity of the habitat, influence the predation of the heteropteran bugs [5, 84, 85] and odonate larvae [46, 86] against dipteran immature. The habitat conditions either augment or reduce the vulnerability of the prey species like tadpoles [85, 87, 88] and larvae of mayfly [73, 74, 89], mosquito [5] and chironomid midge [5]. While the movement of the predators is impeded due to the habitat complexity, the presence of the pebbles and the macrophytes facilitates the evasion of predation by the prey species. As a result of the complex habitat conditions, the intensity and interactions among the predators and the prey change considerably. In the present instance, the consumption of both mosquito and chironomid prey (the shared prey) was higher in simple habitat conditions than in the complex habitat conditions, with pebbles and macrophytes. The presence of the macrophytes and the pebbles lead to the altered interaction among the species involved resulting in reduced number of the dipteran prey mortality reflected in the *k* values (Fig 5). In all instances, irrespective of the density levels of the predator and the prey, the pattern of the prey vulnerability remained the same. For both the mosquito and the chironomid larvae, complex habitat condition lowered the risk of predation while offering newer sites as refuge. Similarly, the efficacy of the heteropteran was lowered due to the presence of the complex habitat conditions, with the movements being impaired due to reduced open space available. However, species specific variation in the prey consumption was also observed among the three predator species (Tables 2 and 4, S1 File).

The mosquito consumption by the insect predators *D*. *rusticus* and *A*. *bouvieri* were significantly reduced under the complex habitat conditions contrast to the simple conditions, when present as single predator [5, 10]. In the present instance similar results were observed with the IGP system involving these predators, reinforcing the concept that the complexity of the habitat conditions influences the prey predator interactions [29, 89–96]. In a similar experimental setup, it was observed that the prey selection by the odonate larvae in an IGP system, is influenced by the habitat conditions, where smaller prey individuals were selectively vulnerable under complex habitat conditions relieving the predation pressure on the larger prey [29]. The vulnerability of the IG prey was complementary to the vulnerability of the shared prey. As shown in Fig 4, the pattern of the vulnerability reduced with the complex habitat conditions but the response was density dependent. With higher density of the shared prey, the IG prey mortality was also reduced considerably reflected in all the habitat conditions. The identity of the top predator was also an important factor due to the differences in the predatory efficacy and the intensity of the predation exhibited by the water bugs contrast to the water stick insect and the water scorpion. Nonetheless, in all instances, the predators consumed higher numbers of the IG prey in complex habitat condition, while the shared prey mortality was higher in the open or simple habitat condition.

It was apparent that the density of the shared prey and the IG prey determines the extent of the risk to predation for the shared prey as well as the IG prey. When shared prey is abundant the mortality of the IG prey was reduced but the extent of reduction varied with the habitat conditions. Although the two factors–habitat conditions, and shared prey density were not tested individually, it is apparent that the trends in the prey mortality under simple habitat conditions contrast to the complex habitat condition are significantly different with higher size effects for density. This is a sole indicator supporting that density had a significantly higher impact on prey mortality than the habitat conditions. In the IGP system involving the heteropteran predators and dipteran prey, the density of the interacting species seems to be more important than the habitat conditions. Thus, irrespective of habitat conditions the mortality of the IG prey will be influenced by the density of the interacting species than the habitat conditions. Perhaps, the insect predators are more adapted to the complex habitat conditions as observed in the natural systems where vegetations and the physical structures seems to govern the habitat quality and arena for interactions of the prey and predator [97–100]. In contrast to the complex habitat conditions the prey consumption remained higher in the simple conditions because the predators could avail greater space for locating and charging the prey species like mosquito. Similarly for the chironomid prey, the open space provides higher chances for being conspicuous to the predators and therefore increases the risk of attack by the predators. The predator specific difference in the outcome of the mosquito and chironomid species mortality was also obvious for the three heteropteran predators both in IGP system and in situations as a single predator. The water bug *D*. *rusticus* is comparatively more active and hunts for the prey items than the nepid predators *R*. *filiformis* and *L*. *griseus*. As a consequence the extent of mortality of the mosquito and chironomid larvae in both single predator and IGP system was higher when *D*. *rusticus* was present in the system. In all instances the compliance of the observed data with the model enables predictions about the possible consequences under natural system when these predators are present together. Extending the observed results for the biological regulation of both the mosquito and chironomid populations, it may be inferred that the vulnerability of the mosquito will depend more with the density of the interacting predators and the habitat condition. Presence of vegetations will favour the mosquitoes while the simple conditions will increase the chances of success of the predators. The mortality for the IG prey will depend on the density of the interacting shared prey and the IG predator, in all habitat conditions, in a reciprocal manner complementary with the mosquito prey mortality.

The mortality of shared prey was highest in simple condition, followed by vegetation only, pebbles only and vegetation and pebbles condition in sequence. The efficacy of the IG predators was noted as *D*. *rusticus* ≥ *L*. *griseus*>*R*. *filiformis* for both shared prey and IG prey mortality. The mortality of *A*. *bouvieri* (IG prey) increased with the increase in complexity with lowest mortality in simple condition, followed by vegetation only, pebbles only and vegetation and pebbles condition in sequence. Earlier studies have demonstrated that the predation on mosquito immature is reduced in structured conditions created by the macrophytes *Pistia stratiotes* and stems of *Jussiaea repens* in case of the water bugs *Diplonychus* sp. [10]. The water bugs *D*. *rusticus*, *R*. *filiformis* and *L*. *griseus* exhibited differential pattern of prey consumption and the prey preferences were significantly reduced under the complex habitat conditions contrast to the simple conditions, when present as single predator [5].

Presence of filamentous algae provides refuge and food to the larvae of the mosquitoes *Anopheles pseudopunctipennis* and reduces the vulnerability to black molly *Poecilia sphenops* in rice field conditions [93]. The prey capture success in pygmy perch *Nannoperca australis* and the damselfly nymphs *Ischnura heterostrica tasmanica*, was not affected by macrophytes, but these predators were less effective in sites with higher structural complexity of habitats [84]. Among insect predators, the prey capture of *Belostoma oxyurum* to tadpoles was reduced in complex habitats. The tadpole prey used the macrophytes as refuge and successfully evaded the attack by the predatory water bug *B*. *oxyurum* [85]. Similar observations were noted with the water bugs *B*. *fluminea* and the odonate nymphs *Anax junius* when sharing the tadpoles of *Bufo terrestris* as prey [101]. Thus it appears that the prey vulnerability is influenced by the presence of the macrophytes and physical elements that render structural complexity and act as refuge. The observations on prey mortality (both IG prey and shared prey) followed a similar trend in the long term study. Both density and habitat effects were prominent in the long term studies in compliance with the findings of earlier experiments. In the long term studies, the decrease in the shared prey mortality (both chironomid and mosquito larvae) dwindled with time as a response to interspecific interactions between the two predators as a part of IGP system. Extending the results of the long term studies on IGP system, it may be assumed that under natural conditions, the shared prey mortality will be affected by the presence of multiple heteropteran predators. In comparison to the single predator system presence of multiple predators involve the interspecific interactions among the predators affecting regulation of the target prey [74–76, 84, 101, 102]. Interspecific interactions between IG predator and IG prey benefits the coexistence of the target prey (mosquito and chironomid larvae) in natural conditions [103].

The aquatic predatory insects are promoted for the regulation of the mosquitoes since long, owing to their abundance in the mosquito larval habitats, like rice fields, temporary pools and allied water bodies [48, 104, 105]. However, being generalist in prey choice, the dietary range of the predatory insects is quite broad which raises questions about the effective regulation of the mosquitoes [5, 106–108]. Among several factors, the prey choice, relative density of the predatory insects [86, 109–112], and the environmental conditions [5, 113] are considered as key factors in accomplishing successful regulation of the mosquitoes. As is known for the mosquito larval habitats, the possibilities of the indirect interactions are considerably high [34], which may reduce the effective regulation of the mosquitoes by the predatory insects. Owing to the wide range of dietary choice and quite high appetite, the predatory insects (Heteroptera) often become a part of the intraguild predation (IGP) [39, 40, 114], which may slow down the process of the mosquito regulation. Empirical studies have shown that mosquito regulation can be affected in IGP involving the water bugs and the backswimmers [18, 55, 56]. The relative density of the shared prey (mosquito or chironomid larvae) [55, 56]and the taxonomic identity and relative density of the IG predator [18] are crucial factors determining the mortality of the target prey. Actually, the consumption of the IG prey (backswimmers) in the low-density level of the target prey reduces the risk to predation of the shared prey. Thus, the role of the IG prey was modulating the strength of interactions and ultimately the vulnerability of the shared prey. Also, the identity of the top predators in the IGP system influenced the outcome of the shared prey (mosquito or chironomid larvae) mortality. Apart from the IGP, the prospective apparent competition among the shared prey and IG prey also reduced the effective regulation of the mosquito larvae.

## Conclusion

In the context of conservation biological control [115], the predatory insects are promoted to regulate the mosquito prey naturally. In comparison to the other modes of biological control, the conservation biological control sustains species diversity in addition to the regulation of the mosquitoes. Considering the species diversity in the rice fields, temporary pools and the allied water bodies [48], the conservation biological control [115] is a feasible option for mosquito regulation. Although the multiple predators and their dietary choice may increase the possibility of evasion by the mosquitoes, the ill effects on the non-target species and the environmental concerns increase the priority of the conservation biological control as an effective measure. However, evaluation of the indirect interactions involving the predatory insects and the mosquito is essential to understand and predict the prospective mosquito species regulation. The use of the generalist insect predators in the biological control has been promoted in several ways to ensure conservation as well as regulation of the mosquitoes [116–118]. Indirect interactions, in contrast, are constraints that impede the successful regulation of the mosquitoes. With IGP as a possibility, the effective regulation of the mosquitoes is affected, which was further reduced in the complex habitat conditions, as observed in the present instance. In complex habitat conditions, the IG prey was consumed at a greater rate than the shared prey, which reduced the vulnerability of the mosquito to the predators. In the background of the habitat complexity, the IGP system involving the insect predators and the mosquito prey provides the possibility under which the predators can reduce the target prey population as well as the situations where the shared prey can evade the predators thereby sustaining the population. Similar inferences can be made for the chironomid larvae, when considered as a shared prey in IGP system. In situations where chironomids are considered as nuisance pest, the larval regulation is recommended. Alternatively, the consumption of the chironomid larvae sustains the population of several predatory insects in the freshwater wetlands. As a result, the population of the predatory insects is maintained in absence of mosquito prey. No doubt that the predatory insects may also consume other prey, but the chironomid larvae are one of the dipteran insects that share many of the mosquito larval habitats and constitute the food for the predatory insects. Further studies regarding preference for the chironomid and mosquito larvae may be carried out to highlight the effective regulation of both the species in natural habitats, where they occur together. Apparently, the habitat conditions determine the prey-predator interactions and therefore the possibility of the effective regulation of the mosquitoes in the rice fields and similar wetlands featured by huge extent of habitat complexity and the predator diversity.

## Supporting information

S1 File The experimental design and the selected results in brief. (DOCX)
