# Supplementary material for: Influence of habitat complexity on the prey mortality in IGP system involving insect predators (Heteroptera) and prey (Diptera): Implications in biological control
Source: PLoS One. 2022 Mar 14;17(3):e0264840. doi: 10.1371/journal.pone.0264840 (PMC8920208; doi:10.1371/journal.pone.0264840)
Supplement: S1 File — (DOCX) [file pone.0264840.s001.docx]

**Supporting information file S1**

**Supporting information caption:** The experimental design and the selected results in brief

| **S1 Table1**: **Outline of Experimental Design:** The outline of the experimental design for determining the effect of habitat condition on the IGP involving heteropteran predators and dipteran prey. | | | |
| --- | --- | --- | --- |
| Parameters | | Details | Remarks |
| **Single predator experiment (Experiment 1)** | | | |
| Predators | *Diplonychus rusticus, Ranatra filiformis, Laccotrephes griseus, Anisops bouvieri* | | Density of 2 and 4 each for first 3 predator species and 10 conspecific individuals of *A. bouvieri* respectively. |
| Prey | Instar IV larvae of *Culex quinquefasciatus* and Chironomid midges | | Density of 50 and 200 for each prey species respectively |
| Habitat | Simple, vegetation, pebbles and pebbles and vegetation (complex). | | 4 levels  Vegetation included the sticks of *Ipomoea aquatica, Pistia stratiotes* and *Vallisneria spiralis* |
| Total Replicates | 1152 (for mosquito larvae) +1152 (for chironomid larvae) | | 18 replicates for each of the predator and prey combinations, with the total being 2304 |
| **IGP system experiment (Experiment 2) (Conspecific IG predators )** | | | |
| IG predator | *Diplonychus rusticus,*  *Ranatra filiformis,*  *Laccotrephes griseus.* | | Density 2 and 4 (for each predator) conspecific individuals |
| IG prey | *Anisops bouvieri* | | Density 10 individuals |
| Shared prey | Instar IV larvae of *Culex quinquefasciatus* or chironomid midge | | Density of 50 and 200 for each prey species respectively |
| Habitat | Simple, vegetation, pebbles and vegetation and pebbles (complex). | |  |
| Total replicates | 864 (for mosquito larvae) +864(for chironomid larvae) | | 18 replicates for each of the predator and prey combinations, with the total being 1728 |
| Prey consumption | Noted for 24 hours | | The number of prey left was counted and deducted from the total given |
| Analysis | Factorial ANOVA on the predation data and multiplicative risk analysis (Vance-Chalcraft and Soluk 2005) followed by post hoc multiple comparison tests;  Multiplicative risk analysis for mosquito and chironomid midges. | | For both IG prey and shared prey |

**S1 Table 2: Consumption of shared prey by top predators:** The consumption (mean ± SE) of shared prey (M, mosquito larvae; C, chironomid larvae) in presence of *D. rusticus* (D), *R. filiformis* (R), and *L. griseus* (L) separately under four different habitat conditions and two levels predator density – (A) 2 predators and (B) 4 predators. (For each predator, n = 18 replicates per prey per habitat condition for each prey type and density)

**A. Predator Density: 2 predators**

| **IG predator only** | | | | | | |
| --- | --- | --- | --- | --- | --- | --- |
| Predator species  **(2 each)** | Prey species | Prey density | Habitat condition | | | |
|  |  |  | simple | vegetation | pebbles | complex |
| L | M | 50 | 38 - 49 41.78 ± .62 | 27 - 41 33.83 ± .93 | 13 - 34 21.33 ±1.42 | 13 - 28 20.94 ±.95 |
| R |  |  | 15 - 27 21.89 ± 1.01 | 8 - 22 14.5± 0.96 | 5 - 16 9.83 ± 0.72 | 4 - 12  7.11 ± 0.58 |
| D |  |  | 22 - 50 38 ± 2.35 | 25 - 41 33.06 ± 1.06 | 14 - 28 21.83 ±0.87 | 9 - 20 13.89 ±0.85 |
| L | C | 50 | 26 - 43 35.5 ± 1.27 | 24 - 40 32.94 ± 0.89 | 9 - 24 17.22 ±0.90 | 18 - 35 28.17 ±1.16 |
| R |  |  | 12 - 38 24.17 ±2.09 | 8 - 18 12.78 ± 0.67 | 5 - 15 9.61 ± .57 | 4 - 14 9.5 ± 0.72 |
| D |  |  | 18 - 36 26.28 ± 1.45 | 15 - 35 23.06 ± 1.41 | 10 - 25 17.17 ±1.09 | 16 - 25 20.11 ±0.59 |
| L | M | 200 | 49 - 92 66.22 ± 3.08 | 33 - 74 50.78 ± 2.4 | 22 - 46 33.39 ±1.68 | 11 - 26 16.72 ±0.99 |
| R |  |  | 58 - 85 71.5 ± 1.95 | 38 - 69 50.06 ± 2.10 | 21 - 47 31.17 ±1.70 | 11 - 25 17.67 ±0.96 |
| D |  |  | 67 - 93 78.61 ± 1.54 | 58 - 86 68.61 ± 2.00 | 20 - 65 46.83 ±3.06 | 35- 53 43.89 ±1.36 |
| L | C | 200 | 45 - 72 59.11 ± 1.62 | 38 - 62 48.39 ± 1.69 | 20 - 45 30.5 ± 1.56 | 28 - 45 36.89 ±1.11 |
| R |  |  | 35 - 56 45.22 ± 1.34 | 27 - 51 34.72 ± 1.46 | 15 - 37 25.39 ±1.48 | 6 - 28 14.11 ±1.64 |
| D |  |  | 51 - 82 68.72 ± 2.25 | 45 - 72 56.11 ± 1.80 | 36 - 46 40.61 ±0.70 | 31 - 51 42.22 ±1.28 |

**B. Predator density: 4 predators**

| IG predator only | | | | | | | |
| --- | --- | --- | --- | --- | --- | --- | --- |
| Predator species  **(4 each)** | Prey species | Prey density | Habitat condition | | | |  |
|  |  |  | simple | vegetation | pebbles | complex |  |
| L | M | 50 | 45 - 50 48.67 ± .39 | 31 - 48 38.78 ±1.07 | 27 - 48 37.5 ± 1.42 | 18 - 35 25.28 ±1.22 |  |
| R |  |  | 25 - 42 35 ± 1.16 | 14 - 28 15.5± 0.78 | 11 - 22 15.5 ± 0.78 | 6 - 18 12.39 ± 0.75 |  |
| D |  |  | 38 - 48 42.17 ± 0.75 | 28 - 38 33.72 ± 0.71 | 10 - 26 17.83 ± 1.21 | 5 - 20 11.39 ± 1.06 |  |
| L | C | 50 | 38 - 49 42.28 ± 0.81 | 19 - 42 32.61 ± 1.45 | 11 - 28 20.56 ± 1.48 | 24 - 35 29.67 ± 0.87 |  |
| R |  |  | 20 - 38 27.78 ±1.27 | 11 - 25 18.67 ± 1.08 | 5 - 21 11.22 ±1 .09 | 8 - 23 14 ± 1.06 |  |
| D |  |  | 33 - 43 38.5 ± 0.75 | 22 - 42 32.94 ± 1.33 | 15 - 32 22.39 ± 1.21 | 16 - 32 23 ± 0.95 |  |
| L | M | 200 | 75 - 106 94.83 ± 1.86 | 58 - 90 75.22 ± 2.14 | 41 - 79 53.83 ± 2.14 | 17 - 41 29.11 ± 1.78 |  |
| R |  |  | 72 - 92 83.4 ± 1.32 | 52 - 68 58.72± 0.97 | 35 - 52 43.5 ± 1.27 | 21 - 36 28.89 ± 1.00 |  |
| D |  |  | 72 - 99 85.22 ± 1.68 | 62 - 78 69.78 ± 1.05 | 43 - 63 54 ± 1.26 | 27- 52 38.33 ± 1.46 |  |
| L | C | 200 | 96 - 120 105.11 ± 1.72 | 61 - 85 75.44 ± 1.49 | 39 - 71 53.44 ± 2.01 | 63 - 78 69.89 ± 1.01 |  |
| R |  |  | 52 - 88 66.5 ± 2.14 | 32 - 52 39.72 ± 1.22 | 19 - 36 25.22 ± 1.14 | 10 - 29 20.16 ± 1.16 |  |
| D |  |  | 84 - 114 97.61 ± 1.20 | 52 - 74 63.17 ± 1.38 | 27 - 51 39.39 ± 2.02 | 27 - 64 45.83 ± 2.80 |  |

**S1 Table 3: Consumption of shared prey by *A. bouvieri:*** The consumption (mean ± SE) of shared prey (M, mosquito larvae; C, chironomid larvae) in presence of ten individuals of *A. bouvieri* as predator separately under four different habitat conditions and two levels of predator density. (For each predator, n= 18 replicates per prey per habitat condition for each prey type and density)

| **10 IG prey** | | | | | |
| --- | --- | --- | --- | --- | --- |
| Prey species | Prey density | Habitat condition | | | |
|  |  | simple | vegetation | pebbles | complex |
| M | 50 | 16 - 35 26.44 ± 1.27 | 12 - 25 19.33 ±0 .95 | 8 - 22 21.33 ± 1.42 | 6 - 20 12.61 ±1.00 |
| C |  | 7 - 22 16.94 ± 0.81 | 10 - 20 13.72 ± 0.68 | 6 - 19 9.61 ± 0.76 | 5 - 16 9.61 ± 0.75 |
| M | 200 | 60 - 95 82.61 ± 2.56 | 42 - 62 52.17 ± 1.23 | 22 - 48 34.06 ± 1.43 | 14 - 38 24 ± 1.46 |
| C |  | 58 - 79 68.11 ± 1.75 | 33 - 61 43.28 ± 1.58 | 21 - 53 38.78 ± 2.33 | 20 - 37 29.22 ± 1.10 |

**S1 Table 4: Mortality of shared prey in IGP system:** The mortality (mean ± SE) of shared prey (M, mosquito larvae; C, chironomid larvae) in heteropteran IGP using *D. rusticus* (D), *R. filiformis* (R), and *L. griseus* (L) separately as IG predators and ten individuals of *A. bouvieri* as IG prey under four different habitat conditions and two levels of predator density. (For each predator, n= 18 replicates per prey per habitat condition for each prey type and density)

**A. 2 IG predators**

| **Predator species** | **Prey species** | **Prey density** | **Habitat condition** | | | |
| --- | --- | --- | --- | --- | --- | --- |
|  |  |  | **simple** | **vegetation** | **pebbles** | **complex** |
| D | M | 50 | 37 - 50 46.67 ±0.91 | 18 - 43 33.78 ± 1.65 | 12 - 41  26.11 ± 2.02 | 8 - 39  22.61 ± 1.85 |
| R |  |  | 8 - 35 20.22 ±1.89 | 9 - 28 18.56 ± 1.40 | 9 - 25  15.94 ± 1.12 | 8 - 27  15.17 ± 1.34 |
| L |  |  | 28 - 42 34.67 ±1.05 | 17 - 32 24.56 ± 1.07 | 13 - 34  21.33 ± 1.42 | 5 - 23  12.56 ±1.06 |
| D | C | 50 | 34 - 45 40.78 ±0.79 | 20 - 40 29.94 ± 1.20 | 14 - 27  20.17 ± 0.86 | 7 - 20  12.05 ± 0.75 |
| R |  |  | 23 - 36 29.44 ±0.74 | 19 - 31 24.28 ± 0.96 | 9 - 27  15.94 ± 1.1 | 7 - 14  9.78 ± 0.48 |
| L |  |  | 37 - 48 41.67 ±0.91 | 29 - 41 35.67 ± 0.89 | 18 - 38 26.17 ± 1.27 | 7 - 25  14.89 ± 1.07 |
| D | M | 200 | 116 - 185 151.94±4.66 | 94 - 154 122.28 ±4.03 | 56 - 129 94.39 ± 3.92 | 78 - 125  97.33 ± 3.10 |
| R |  |  | 115 - 165 138.28 ±2.18 | 85 - 129  110 ± 3.11 | 64 - 107 87.72 ± 2.82 | 52 - 89  71.28 ± 2.82 |
| L |  |  | 124 - 167 146.11 ±2.83 | 95 - 148 120.28 ±3.17 | 65 - 120 96.28 ± 3.6 | 56 - 102  73 ± 3.03 |
| D | C | 200 | 116 - 165 144.78 ±3.46 | 102 - 150 122.78 ±2.80 | 85 - 125 103.67 ±3.27 | 75 - 125  96.89 ± 3.89 |
| R |  |  | 51 - 88 70.78 ± 2.48 | 42 - 65 52.39 ± 1.65 | 28 - 61 39.67 ± 2.24 | 18 - 36  26.06 ± 1.28 |
| L |  |  | 100 - 150 127.22 ±3.36 | 95 - 137 119.94 ±2.80 | 79 - 140 101.5 ± 3.75 | 41 - 92  67.55 ± 2.94 |

**B. 4 IG predators**

| **predator species** | **Prey species** | **prey density** | **Habitat condition** | | | |
| --- | --- | --- | --- | --- | --- | --- |
|  |  |  | **simple** | **vegetation** | **pebbles** | **complex** |
| D | M | 50 | 47 - 50  49.72 ± 0.19 | 28 - 50  42 ± 1.51 | 18 - 44  32 ± 1.60 | 20 - 35 26.56 ±.13 |
| R |  |  | 17 - 42  30.22 ± 1.74 | 17 - 31  22.83 ± 0.94 | 7 - 25  13.78 ± 1.28 | 7 - 17 11.5 ± 0.62 |
| L |  |  | 46 - 50  48 ±.28 | 17 - 31 22.83 ±0.94 | 7 -25  12.78 ±1.29 | 7 - 17 11.5 ±0.62 |
| D | C | 50 | 44 - 49  46.33 ± 0.40 | 31 - 45 38.89 ± 0.89 | 19 - 36 19.17 ± 1.50 | 9 - 30 19.17 ±1.50 |
| R |  |  | 29 - 40  35.33 ± 0.74 | 15 - 33 22.11 ± 1.18 | 15 - 29 20.22 ± 0.83 | 6 - 19 13.06 ±0.82 |
| L |  |  | 40 - 50  47.56 ± 0.52 | 30 - 46  38 ± 0.98 | 18 - 41 29.56 ± 1.37 | 8 - 33 16.89 ±1.48 |
| D | M | 200 | 164 - 199  190.17 ± 2.10 | 156 - 195 175.78 ± 2.45 | 105 - 178 151.61 ± 4.66 | 98 - 145 126.06 ±3.31 |
| R |  |  | 129 - 179  158.83 ± 3.33 | 121 - 160 139.78 ± 2.55 | 97 - 141 118.61 ± 3.02 | 59 - 109 83.61 ± 3.11 |
| L |  |  | 161 - 195  182.6 ± 4.2 | 126 - 182 152.89 ± 3.81 | 100 - 150 127.33 ± 2.97 | 65 - 115 94.17 ±2.92 |
| D | C | 200 | 159 - 189  174.56 ± 2.12 | 99 - 154 129.17 ± 3.30 | 92 - 118 105.28 ± 1.82 | 62 - 112 84.83 ± 3.70 |
| R |  |  | 135 - 159  144.83 ± 1.46 | 105 - 157 128.17 ± 2.94 | 108 - 140 124.11 ± 2.21 | 52 - 93 70.56 ± 3.26 |
| L |  |  | 159 - 190  177.89 ± 1.80 | 138 - 165 150.22 ± 1.10 | 100 - 132 116.67 ± 2.28 | 62 - 102 84.56 ± 2.59 |

**S1 Table 5: Mortality of IG prey (*A. bouvieri*) in IGP system:** The mortality (mean ± SE) of *A. bouvieri* (IG prey) in heteropteran IGP using *D. rusticus* (D), *R. filiformis* (R), and *L. griseus* (L) separately as IG predators and ten individuals of *A. bouvieri* as IG prey and two density levels (50 and 200) of shared prey (M, mosquito larvae; C, chironomid larvae) under four different habitat conditions and two levels predator density (A, 2 IG predators; and B, 4 IG predators). (For each predator, n= 18 replicates per prey per habitat condition for each prey type and density)

**A. Predator density – 2 predators**

| **Predator species (2 each)** | **Prey species** | **Prey density** | **Habitat condition** | | | |
| --- | --- | --- | --- | --- | --- | --- |
|  |  |  | **simple** | **vegetation** | **pebbles** | **complex** |
| D | M | 50 | 0 - 4 2.22 ± 0.26 | 0 - 6 2.78 ± 0.45 | 2 - 6 3.22 ± 0.29 | 0 - 7 4.22 ± 0.36 |
| R |  |  | 0 - 4  2.06 ± 0.32 | 1 - 4 2.78 ± 0.24 | 2 - 6 3.83 ± 0.29 | 2 - 7 4.17 ± 0.34 |
| L |  |  | 0 - 2 0.61 ± 0.14 | 0 - 4 2.5 ± 0.32 | 0 - 5 3.11 ± 0.29 | 2 - 6 4.17 ± 0.27 |
| D | C | 50 | 0 - 6  3.33 ± 0.38 | 1 - 6 3.44 ± 0.33 | 3 - 7 5 ± 0.30 | 0 - 8 4.78 ± 0.45 |
| R |  |  | 2 - 7 3.44 ± 0.35 | 2 - 6 3.39 ± 0.31 | 3 - 8 5.06 ± 0.31 | 4 - 10 6.44 ± 0.38 |
| L |  |  | 1 - 4 2.39 ± 0.23 | 0 - 4 2.17 ± 0.28 | 2 - 6 3.28 ± 0.28 | 0 - 7 3.78 ± 0.42 |
| D | M | 200 | 0 - 2 0.56 ± 0.17 | 0 - 4 2.33 ± 0.29 | 1 - 6 3.17 ± 0.34 | 3 - 6 4.72 ± 0.25 |
| R |  |  | 0 - 3 1.11 ± 0.21 | 1 - 3 2.33 ± 1.18 | 1 - 6 3.33 ± 0.34 | 3 - 7 4.06 ± 0.26 |
| L |  |  | 0 - 2 0.44 ± 0.15 | 0 - 4 1.78 ± 0.33 | 0 - 4 2.56 ± 0.27 | 2 - 7 4.17 ± 0.30 |
| D | C | 200 | 0 - 3 1.22 ± 0.24 | 0 - 5 2.22 ± 0.32 | 0 - 7 3.33 ± 0.44 | 2 - 9 4.61 ± 0.39 |
| R |  |  | 0 - 6 2.56 ± 0.35 | 0 - 5 2.94 ± 0.27 | 0 - 6 3.22 ± 0.33 | 3 - 7 4.83 ± 0.28 |
| L |  |  | 0 - 3 1.11 ± 0.21 | 0 - 5 2.11 ± 0.32 | 1 - 5 2.78 ± 0.26 | 3 - 9 4.94 ± 0.37 |

**B. Density – 4 predators**

| **Predator species (4 each)** | **Prey species** | **Prey density** | **Habitat condition** | | | |
| --- | --- | --- | --- | --- | --- | --- |
|  |  |  | **simple** | **vegetation** | **pebbles** | **complex** |
| D | M | 50 | 0 - 2 1 ± 0.18 | 1 - 7 2.78 ± 0.34 | 2 - 5 3.44± 0.25 | 4 - 7 5.55 ± 0.18 |
| R |  |  | 2 - 5 3 ± 0.23 | 1 - 6 3.61 ± 0.33 | 2 - 8 5.94 ± 0.41 | 4 - 9 7.27 ± 0.28 |
| L |  |  | 0 - 3 1.33 ± 0.23 | 1 - 5 2.55 ± 0.23 | 2 - 5 3.61 ± 0.23 | 5 - 8 6.67 ± 0.23 |
| D | C | 50 | 0 - 3 1.89 ± 0.24 | 0 - 4 2.11 ± 0.29 | 1 - 6 3.11 ± 0.30 | 3 - 7 5.11 ± 0.24 |
| R |  |  | 0 - 4 2.11±0.25 | 2 - 5 3.11 ± 0.21 | 2 - 8 4.06 ± 0.37 | 2 - 9 4.5 ± 0.39 |
| L |  |  | 1 - 4 2.83 ± 0.20 | 2 - 5 3.5 ± 0.23 | 2 - 5 3.67 ± 0.23 | 2 - 7 4.89 ± 0.33 |
| D | M | 200 | 0 - 2 0.56 ± 0.17 | 1 - 4 2.56 ± 0.18 | 1 - 6 3.44 ± 0.32 | 2 - 7 4.28 ± 0.30 |
| R |  |  | 0 - 3 1.44 ± 0.23 | 1 - 5 3.55± 0.26 | 2 - 8 5.28 ± 0.37 | 4 - 10 6.17 ± 0.39 |
| L |  |  | 0 - 2 0.78 ± 0.17 | 1 - 5 2.83 ± 0.30 | 2 - 7 4.72 ± 0.30 | 4 - 8 5.94 ± 0.32 |
| D | C | 200 | 0 - 2 0.83 ± 0.20 | 0 - 4 1.83 ± 0.31 | 1 - 5 2.89 ± 0.25 | 0 - 6 3.56 ± 0.36 |
| R |  |  | 0 - 2 0.72 ± 0.18 | 0 - 4 2.33± 0.28 | 1 - 6 3.56 ± 0.34 | 2- 7 4.17 ± 0.32 |
| L |  |  | 0 - 2 1.06 ± 0.17 | 2 - 5 3 ± 0.23 | 1 - 6 3.61± 0.32 | 2- 7 4.55± 0.32 |
